# In-Depth Comparison of Dry Particle Coating Processes Used in DPI Particle Engineering

**DOI:** 10.3390/pharmaceutics13040580

**Published:** 2021-04-19

**Authors:** Nicholas Bungert, Mirjam Kobler, Regina Scherließ

**Affiliations:** 1 Department of Pharmaceutics and Biopharmaceutics, Kiel University, Grasweg 9a, 24118 Kiel, Germany; nbungert@pharmazie.uni-kiel.de; 2 Meggle Excipients and Technology, 83512 Wasserburg, Germany; mirjam.kobler@meggle.de

**Keywords:** dry powder inhalation, compound excipient, force control agent, surface energy, adhesion strength

## Abstract

High-shear mixer coatings as well as mechanofusion processes are used in the particle-engineering of dry powder inhalation carrier systems. The aim of coating the carrier particle is usually to decrease carrier–drug adhesion. This study comprises the in-depth comparison of two established dry particle coating options. Both processes were conducted with and without a model additive (magnesium stearate). In doing so, changes in the behaviour of the processed particles can be traced back to either the process or the additive. It can be stated that the coarse model carrier showed no significant changes when processed without additives. By coating the particles with magnesium stearate, the surface energy decreased significantly. This leads to a significant enhancement of the aerodynamic performance of the respective carrier-based blends. Comparing the engineered carriers with each other, the high-shear mixer coating shows significant benefits, namely, lower drug–carrier adhesion and the higher efficiency of the coating process.

## 1. Introduction

The increasing prevalence of respiratory diseases has been reported frequently [1]. Due to numerous advantages of dry powder inhalation (DPI) formulations, their importance has increased in the past decades. Out of several formulation options, the interactive blend is the most important one when it comes to frequency of use [2]. To reach processability of higher cohesiveness, micronised drug particles are mixed with a coarse carrier. While mixing, the drug attaches to the carrier but just so strong that it can detach in the inhalation airstream. Commercially available respiratory medicines show insufficient drug delivery to the lungs [3,4], usually significantly below 50%. The share of the active pharmaceutical ingredient (API), which is not able to detach and disperse, will impact with the carrier in the throat or in the upper airways. This can lead to side effects and decreased API concentrations in the deeper lung tissues. There are numerous strategies of using additives for the improvement of aerodynamic performance of carrier-based blends. Beside the addition of lactose fines [5], magnesium stearate [6] is a well-established additive as a force control agent (FCA). Several scientific articles report about the beneficial effect of the addition of the well-known lubricant [6,7]. Nearly as many articles try to explain the underlying principle of the enhanced aerodynamic performance [8,9]. To date, the operating principle is not completely clarified. It is most likely a combination of smoothening and weakening of binding energy. Since Hertel et al. (2020) found that force control agents combined with additional extrinsic fines do not lead to the addition of beneficial effects, the decrease in binding energy using FCAs is further substantiated. This is due to the fact that additional fines work, amongst other interactions, through the occupation of higher energy sites on the carrier. Weakening those makes additional fines superfluous, as also observed by Hertel et al. In the past, different approaches were published describing how magnesium stearate is added and the corresponding impact of it. The most common attempt is just by blending the carrier and additive in a high-shear mixer. Recent work showed an evenly distributed lubricant coating for high-shear mixing with magnesium stearate using TOF-SIMS (time-of-flight secondary-ion mass spectrometry) [10]. Another strategy is to make use of the so-called mechanofusion [11], an approach that merges substances with high mechanical energy input. The claimed benefit is a mechano-chemical reaction in the contact area of two solid substances leading to new compositions [12]. While being processed, the sample in the mechanofusion reactor is compressed and simultaneously stressed by intense shear. Both dry particle coating strategies are dependent on rotor speed and processing time. The parameters in this work are based on already published data using these methods [7,11,13]. The comparison between the high-shear and low-shear addition of the lubricant has already been investigated in the past, showing no significant increase in aerodynamic performance after low-shear mixing [14]. In general, numerous methods can be used for dry particle coatings, even though not all of them are suitable for coating DPI carrier particles without fundamental changes in particle morphology. A wide range of applications had been extensively reviewed recently by Sharma et al. [15]. It is reported that mechanofusion with FCAs leads to enhanced flowability and powder de-agglomeration combined with an overall decreased and more homogeneous surface energy of the created compound particles [13,16,17]. The surface energy of solids is undoubtedly a powerful parameter to characterise a particles’ interaction behaviour. However, since it is able to detect even small changes in particle properties introduced by processing (e.g., milling, amorphisation, recrystallisation), how much of the resulting changes observable after the dry particle coating are either additive or solely process induced is a justified question.

In this study, an assessment of additive-free and additive-containing processed powders was conducted. This enables the comparison of both approaches on different levels, allowing conclusions on whether changes can be attributed to the additive or the process itself. Since both methods are already established and lead to distinct increases in fine particle fraction (FPF), it is important to investigate the differences. Moreover, this study can help in deciding which particle coating process shall be preferred for adding magnesium stearate or similar additives.

## 2. Materials and Methods

### 2.1. Materials

As the model carrier in the coating processes, InhaLac^®^ 230 (Meggle, Wasserburg, Germany) a crystalline, sieved inhalation grade lactose monohydrate was used. Magnesium stearate (Parteck^®^ LUB MST, Merck, Darmstadt, Germany) served as the model coating material. For the sake of simplicity, the materials are shortened to the following: InhaLac 230 (IH230), magnesium stearate (MgSt), InhaLac 230 processed in the mechanofusion reactor (IH230AMS, suffix “+” or “−” indicating if processed with the additive or without), InhaLac 230 processed in the high-shear mixer (IH230HSM, suffix “+” or “−” indicating if processed with the additive or without). Micronised ipratropium bromide served as the model drug (d_90_ < 5 µm, Boehringer Ingelheim Pharma AG & Co. KG, Ingelheim, Germany).

### 2.2. Dry Particle Coating 

The mechanofusion process was conducted with the Angmill Mechanofusion System (AMS) unit of the Picobond^®^ using the Picoline^®^ platform (Hosokawa Alpine, Augsburg, Germany). The carrier and coating materials (2% (*w*/*w*) MgSt) were premixed in a Turbula^®^ blender (Willy A. Bachofen Maschinenfabrik, Basel, Switzerland) at 42 rpm for 5 min. Pre-blends were then transferred to the AMS. The AMS coating started by increasing the rotor speed up to 4000 rpm within one minute and keeping it constant at 4000 rpm over 10 min.

The high-shear mixer (HSM) coating was conducted using the Picomix^®^ unit of the Picoline platform. A rotor speed of 500 rpm was kept constant for 15 min.

All raw materials were sieved before processing (mesh size: 250 µm), as well as the coated samples after processing. To assess the energy input to the powder, the data transfer tool of the Picoline was used. The instrument performance over the whole processes using the same powder mass was averaged and then multiplied with the respective process duration to obtain the total energy input. The procedure was then repeated without powder. The idle running energy was subtracted from the total energy input to calculate the energy input into the powder sample. All coatings were conducted at monitored relative humidity (RH) of 45% ± 15% and room temperature (20 °C to 25 °C).

### 2.3. Determination of Thermal Properties—Dynamic Scanning Calorimetry (DSC)

A power compensation DSC (PerkinElmer DSC 7, PerkinElmer Inc., Waltham, MA, USA) was used for each measurement, and data were evaluated with the PYRIS™ Software (PerkinElmer Inc.). Approximately 5 mg of the respective sample was weighed into an aluminium pan, which was sealed and pierced afterwards to allow gas to escape. A pierced, empty aluminium pan was used as reference. During analysis, the sample was flushed with nitrogen to avoid any oxidation processes. All samples were heated at a rate of 10 °C/min up to 240 °C. Results are plotted as the heat flow (mW) over temperature. Furthermore, the thermogravimetric properties of the sample were analysed (Simultaneous Thermic Analyzer 6000, STA), using the same heating rate and heating range as that of the DSC. This provides additional information on what happens at the events observable in the DSC (e.g., loss of crystal water).

### 2.4. Determination of Crystal Properties—Vapour Sorption Technique

Dynamic vapour sorption (DVS) was carried out using the DVS Resolution (Surface Measurement Systems Ltd., London, UK). For these experiments, approximately 70 mg of the respective sample was weighed into the sample pan. Measurements were carried out at 25 °C. The method consisted of two cycles of increasing the partial pressure of bidistilled water (aq. bidest.) from 0–90% in 10% steps and returning to 0%. At the beginning of every measurement, the sample was conditioned for 180 min at 0% RH. Every step of the method was not limited by time but by a change in mass of below 0.005%/min.

### 2.5. Particle Size Distributions (PSD)

Laser diffraction was applied to determine the particle size distribution (PSD) of the initial and processed material. For this, a Helium Neon Laser Optical System (HELOS^®^, Sympatec GmbH, Clausthal-Zellerfeld, Germany) was equipped with a RODOS^®^ dispersion module and a R4 lens with a measuring range of 0.5 to 350 µm. With compressed air (2 bar), the automatically fed (VIBRI^®^ dry dosing system, Sympatec GmbH) material was dispersed in an aerosol jet towards the measuring zone.

Evaluation of the size distribution was performed with the Windox 5 software (Sympatec GmbH) based on the Fraunhofer enhanced equation.

### 2.6. Specific Surface Area (SSA) and Surface Energy (SE)

The assessment of specific surface area (SSA) using the BET method and the surface energy of solids (SE) was carried out using the surface energy analyser (SEA, Surface Measurement Systems Ltd., London, UK). The powder samples were weighed into silanised glass columns (4 mm inner diameter) and fixed with silanised glass wool on both ends. To avoid hollows and cracks in the powder bed, the filled columns were compressed by tapping for 10 min using the SMS column packer accessory. Prior to every measurement, all columns were conditioned at 0% RH and 10 sccm carrier gas flow (nitrogen) for one hour to get rid of any volatile contamination. For SSA measurements, a series of octane injections was performed, providing an adsorption isotherm. The calculation of the SSA was based on the linear section of the adsorption isotherm (p/p^0^ 0.05–0.35).

The measurement of SE distributions was conducted by injecting a series of alkanes (octane—undecane) for dispersive SE as well as chloroform and toluene for determination of acid-base surface properties. All injected concentrations were based on the respective monolayer capacity, leading to surface coverages from 0.5% up to 10%. For the determination of the dead volume, a double injection of methane was performed at the beginning and at the end of every experiment. All experiments were carried out at 0% RH and 30 °C with a carrier gas flow of 10 sccm.

The raw data were analysed using the SEA Analysis Software (Surface Measurement Systems Ltd., London, UK). Calculations were based on the DellaVolpe scale in combination with the Dorris and Gray approach and the polarisation method [18,19]. For SSA calculations, peak maxima were used, for SE calculations served the peak centre of mass.

### 2.7. Particle Imaging

The imaging of particle morphology was conducted with scanning electron microscopy (SEM) using Phenom XL (Phenom-World BV, Eindhoven, The Netherlands). To guarantee sample grounding and to minimise charging effects, samples were fixed onto carbon stickers and gold-sputtered with BAL-Tec SCP 050 Sputter Coater (Leica Instruments, Wetzlar, Germany). All images were taken at 1000× or 2500× magnitude with an acceleration voltage of 10 kV and with the use of a backscatter detector.

### 2.8. High-Performance Liquid Chromatography

The quantification of drug content was carried out using high-performance liquid chromatography (Waters Corporation, Milford, MA, USA). The analytical procedure was based on a cyanopropyl-substituted stationary phase (LiChrospher^®^ 100 CN, Merck), using a solvent mixture (71% aq. bidest., 29% acetonitrile, 1.42 g/L hepantesulfonic acid) adjusted to pH 3.2 as mobile phase. The method was validated in terms of system suitability, specificity, precision, repeatability and linearity. The limit of quantification was calculated based on the corresponding ICH guideline (CPMP/ICH/381/95) as 0.08 µg/mL. The quantification calculations were based on an external standard calibration curve (R^2^ > 0.99) covering a concentration range from 0.21 µg/mL to 104.8 µg/mL. All values used were within the calibrated range. All solvents used were chromatographic grade and supplied by Honeywell Riedel-de Haën (Chromasolv, Seelze, Germany).

### 2.9. Preparation of Interactive Blends

All adhesive mixtures were prepared (1% *w*/*w* active ingredient) using the Picomix high-shear mixer module with two mixing steps at 500 rpm and one sieving step (mesh size: 250 µm) in between. All mixing steps were conducted at a monitored RH of 45% ± 15% and room temperature (20 °C to 25 °C). Every mixture comprised a batch size of 30 g and was tested for homogeneity by analysing 10 randomly picked samples via high performance liquid chromatography. A blend was considered homogeneous at a relative standard deviation below 5% and a recovery of 90–110%.

### 2.10. Aerodynamic Assessment

The aerodynamic assessment was carried out using the Novolizer^®^ (MEDA Pharma GmbH & Co. KG, Bad Homburg, Germany) with the fast-screening impactor (FSI, Copley Scientific, Nottingham, United Kingdom). The FSI allows rapid determination of fine particle dose < 5 µm (FPD) and fine particle fraction (FPF). All experiments were operated at a flow rate corresponding to a 4 kPa pressure drop over the inhaler (78.3 L/min). All results are shown as an average of five measurements with five doses per measurement. All FSI experiments took place in a climate chamber with constant environmental conditions of 21 °C and 45% RH. High performance liquid chromatography (Waters Corporation, Milford, MA, USA) was used for drug quantification.

### 2.11. Carrier–Drug Adhesion Screening

For the adhesion strength screening, an e200LS air-jet sieve (AJS; Hosokawa Alpine, Augsburg, Germany) was used. A specified mesh-sized analytical sieve was inserted (20 µm for adhesion strength screening). For every repetition, 5 g of sample was placed on the sieve, covered with a lid and then dispersed via a rotating pressurised air nozzle. A negative pressure of 4 kPa was created by a pump, sucking particles below 20 µm through the sieve. The process was stopped at 6 s, 30 s, 1 min, 2 min, 5 min and 10 min for sampling. Per sampling time, 10 randomised powder samples were picked from above the sieve. From each of the resulting 60 samples, a specific mass of 7 mg (±0.5 mg) was weighed into an Eppendorf tube, dissolved in 2 mL of solvent (aq. bidest.) and analysed in the high-performance liquid chromatography. The resulting drug content was put in in relation to the nominal drug content of the adhesive mixture (namely 1% *w*/*w*). All results are displayed as the average of three measurements at a monitored temperature of 20 °C (±5 °C) and 45% (±15%) RH.

### 2.12. Removal of Residual Excipient Particles

For the removal of residual MgSt particles from the carrier, the e200LS air-jet sieve with a mesh size of 32 µm was utilised. The sieve step was conducted for 15 min with a negative pressure of 4 kPa.

### 2.13. Quantification of Magnesium Stearate

The content of MgSt was measured using atomic absorption spectroscopy (AAS). All samples were dissolved using diluted nitric acid in combination with sonication (20 min) and shaking (15 min). After centrifugation (10 min, 7000 rpm), the samples were diluted and measured using a flame atomisation (air–acetylene flame) AAS system (AAS 3030, PerkinElmer Inc., Waltham, MA, USA). The calculation of the content was based on a six-point calibration curve from zero up to 1 ppm magnesium concentration.

### 2.14. Statistical Evaluation

All statistical calculations were carried out with Microsoft Excel 2016 (Microsoft Corporation, Redmond, WA, USA).

## 3. Results and Discussion

The in-depth investigation of two established dry particle coating processes was conducted with a broad spectrum of physico-chemical analysis tools. Since those particle coating processes are used for formulation optimisation attempts [7,11] in the dry powder inhalation area, an aerodynamic assessment of the respective interactive blends was also part of this study. Both coating mechanisms (AMS, HSM) are based on the introduction of mechanical stress: on the one hand, utilising a rotating stirring tool, on the other hand, forcing the powder through a tight gap.

The energy input in both preparation procedures was determined by recording the performance data of the instrument for both conducted processes using the same sample mass. The energy input into the powder was calculated to be 1234 J for the AMS and 1318 J for the HSM process. This corresponds to approximately 6% higher energy input in the HSM coating attempt. Even though both processes show no major differences in energy input, both methods introduce considerable amounts of mechanical energy into the sample. As a comparison: preparing an interactive blend as described introduces approximately 250 J of mechanical energy. Since the mechanical stress during dry particle coating could already cause fundamental changes in the particle properties, such as amorphisation or fragmentation, both processes were conducted with and without additives. This allows the evaluation of whether changes in powder behaviour can be attributed to processing or to the additive itself.

### 3.1. Process-Induced Particle Alteration

**Figure 1:**
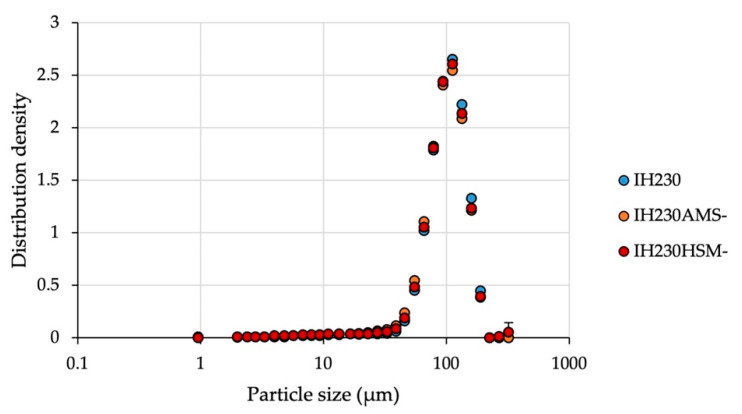
Particle size distribution density of the raw material in comparison to processed powders without the additive. *n* = 3, error bars show SD.

When applying mechanical stress to a powder sample, an expectable change is in particle size distribution. To make sure no particle fragmentation occurs, particle size distributions were measured via laser diffraction. The results are displayed in Table 1 and Figure 1.

The results demonstrate that even though considerable mechanical stress was applied during both processes, the particle size distribution is unchanged, which indicates that particles were not fragmented significantly when processed without the additive.

Since particle size distributions have a huge impact on the performance as a carrier platform in dry powder inhalation formulations [20], not decreasing particle sizes is very important for a meaningful comparison. In addition to particle size distribution measurements, octane adsorption isotherms were determined to calculate the specific surface area. The BET data confirmed that no particle fragmentation occurred (Table 2).

Otherwise, one would observe increasing surface areas after processing due to decreased particle sizes.

**Figure 2:**
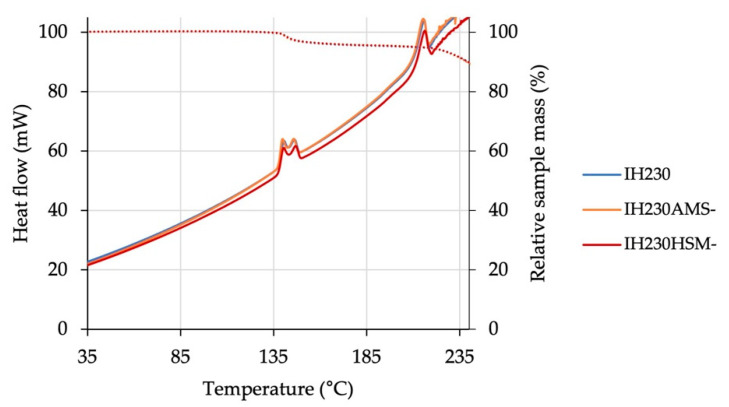
Results of DSC analysis of the raw material in comparison to processed powders without the additive. The secondary y-axis shows the relative mass decrease (dotted lines). *n* = 3. Negligible variation between the compared data series results in overlapping curves.

Another alteration, which could have been introduced even without particle fragmentation, is the formation of crystal defects, leading to partial amorphisation. Since the starting material, InhaLac 230, is a highly crystalline lactose, even small amounts of process-induced amorphous content are detectable. As analytical tools, two different methods were used: differential scanning calorimetry (DSC) in combination with thermogravimetric analysis (TGA) and dynamic vapour sorption (DVS). Both techniques were applied to the additive-free samples. Thus, solely the process-induced changes in solid state properties can be tracked. All DSC graphs (Figure 2) show essentially the same peaks: one endotherm double peak starting at almost 140 °C represents the dehydration of the lactose monohydrate, the other endotherm peak, beginning at 200 °C, indicates the decomposition and charring of the anhydrous lactose samples. Neither a glass transition step nor a recrystallisation peak indicating amorphous content was observed in any plot. A combination of DSC and thermogravimetric measurements allows for further insights into thermal events by analysing temperature-related mass changes. The step at 140 °C was aligned with a relative mass decrease of approximately 5%, which represents the loss of crystal water of the lactose monohydrate. The melting and charring at 200 °C led to an unspecific mass decrease.

**Figure 3:**
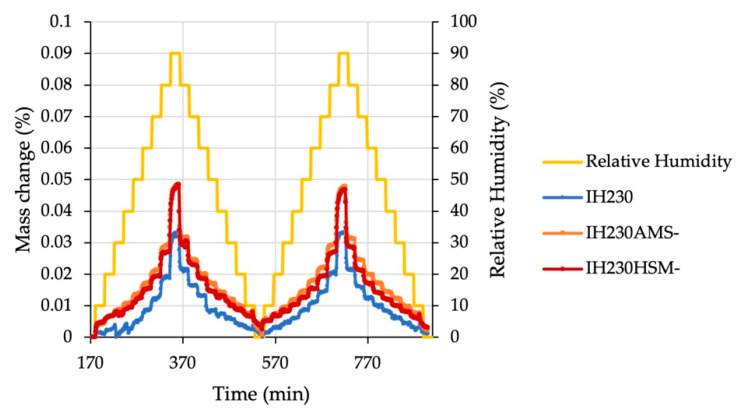
DVS plot showing relative mass change related to the reference mass in dependence of the relative humidity. Sample conditioning data (0–170 min) are not shown.

DVS was chosen as an orthogonal method to verify the conclusion made based on the DSC experiments. For comparability, only additive-free, processed lactose samples were investigated. Figure 3 displays mass changes of all samples. The maximum mass change was approximately 0.05% for all samples unrelated to processing. If recrystallisation of amorphous parts would have occurred, the mass change in the second half of the double cycle would show decreased mass changes. This would be due to less amorphous content (recrystallised in the first half of the cycle), which is able to absorb water and leads to a distinct increase in sample mass. For the investigated samples, this cannot be deduced from the measured values. Furthermore, the relative mass change in general was extremely small, indicating (for a hydrophilic substance, such as lactose) a highly crystalline and non-hygroscopic material.

A slight difference between processed and non-processed samples can be seen. The processed samples reproducibly gained more mass than the non-processed, which can be explained by little differences in surface appearance or the creation of non-recrystallisable defects, which is substantiated by no identifiable differences between both cycles. This combination of methods showed that in both used dry particle coating methods, no introduction of major changes in the solid state of the particles had occurred. This is important not only for stability issues but also for tracing back new particle properties after the process with the additive—not to the process itself, but to the additive.

### 3.2. Additive-Induced Particle Alteration

**Figure 4:**
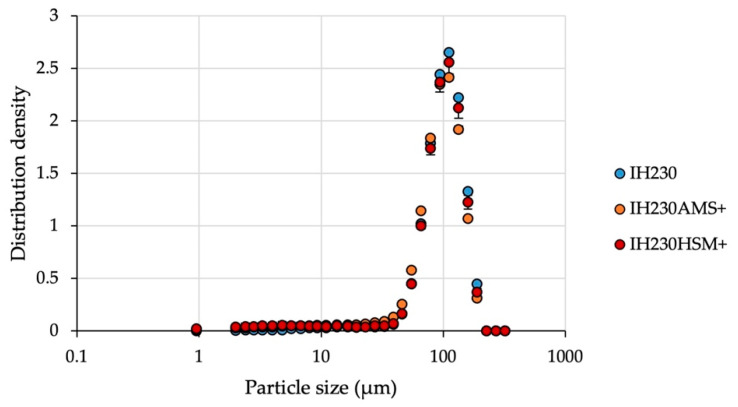
Particle size distribution density of the raw material in comparison to processed powders with the additive. *n* = 3, error bars show SD.

Following the investigation of the addition of mechanical stress, the determination of particle specifications, namely PSD (Figure 4 and Table 1) and BET (Table 2), after processing with the additive was conducted. Since fragmentation or milling of the lactose carrier can be excluded as discussed above, the PSD shift to slightly lower particle sizes (Table 1) can be explained by residual particles of MgSt, which are the size of approximately 6 µm. When the total amount of coating material had not been coated successfully, some loose particles were left in the powder bed and caused significant shifts towards lower PSDs (*p*-value > 0.05 when comparing d_10_, d_50_ and d_90_ values). 

**Figure 5:**
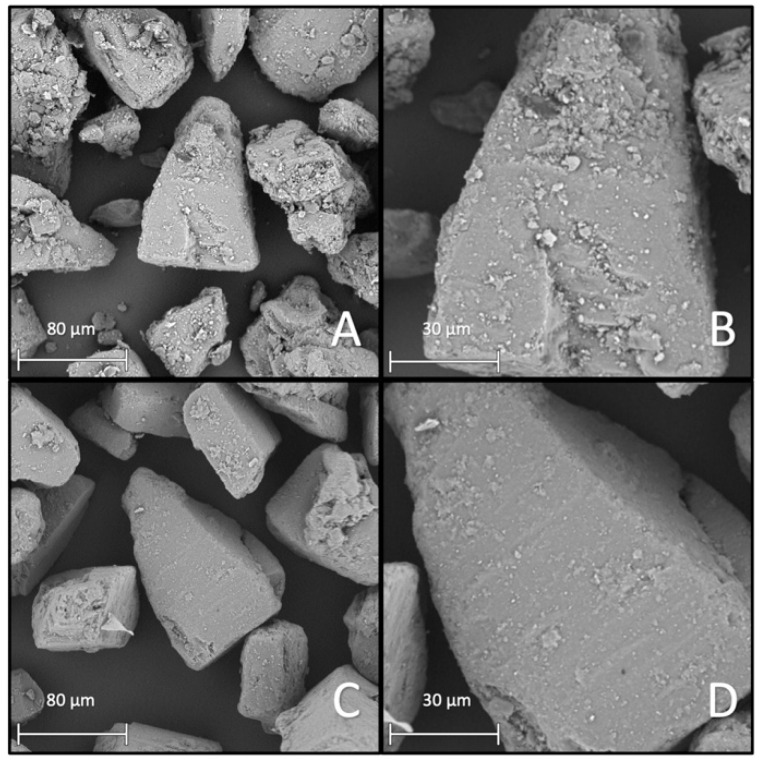
SEM images showing IH230AMS+. (**A**,**B**) Before and (**C**,**D**) after removing residual particles via air-jet sieving. (**A**,**C**) In 1000× magnification and (**B**,**D**) in 2500×. magnification.

**Figure 6:**
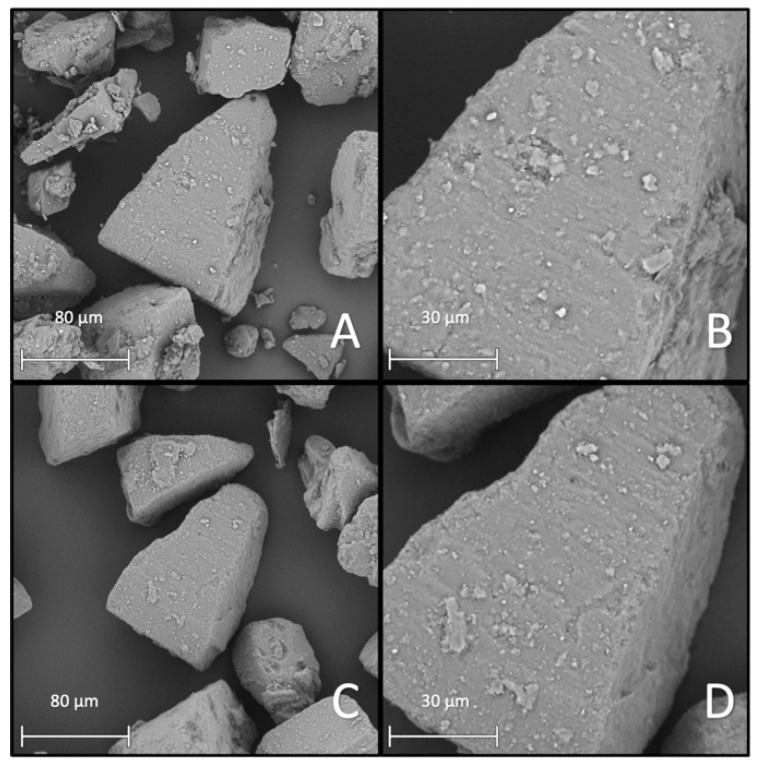
SEM images showing IH230HSM+. (**A**,**B**) Before and (**C**,**D**) after removing residual particles via air-jet sieving. (**A**,**C**) In 1000× magnification and (**B**,**D**) in 2500× magnification.

This observation was supported with the specific surface area results. Both processed samples showed a difference between the BET-specific surface area depending on if processed with or without the additive. The specific surface area of the HSM sample increased to 0.8 m^2^/g while the AMS sample only increased to 0.4 m^2^/g. A general increase in surface can be substantiated by the SEM images (A and B) in Figure 5 and Figure 6. Figure 5A,B show the surface of the AMS sample and Figure 6A,B the surface of the HSM sample. Based on the SEM images, one would assume that the AMS sample should exhibit a larger surface area due to a higher number of small particles (Figure 5B). On the other hand, in Figure 6B, the merging process of the additive and the carrier seemed further developed, with less individual particles on the surface. For further investigation of the coating uniformity, residual fine MgSt particles were removed using the air-jet sieve (mesh size: 32 µm). The resulting particle surfaces are shown in Figure 5C,D and Figure 6C,D, respectively. Comparing the surfaces after removing residual MgSt particles, the high-shear mixed sample looked more similar to the surface before air-jet sieving. For the mechanofused surface, a significant decrease in the number of small particles was observable.

To gain further insight into the coating effectivity, the amount of successfully attached and non-removable MgSt was determined using AAS. In order to assess the share of the strongly bonded additive, the MgSt content in the blend before and after the removal of residual particles was measured. The decrease in percent provides information about the share of the additive, which was not merged but only adhered to the surface after processing. Initially, 2% (*w*/*w*) MgSt was added in both coating strategies. The measured MgSt content of 1.31% and 1.23% before air-jet sieving indicates a loss of more than 30% due to processing. After air-jet sieving, the additive content of the HSM sample decreased by 18.0%, while the content of the AMS sample decreased by over 60% (Table 3). This substantiates the observations of the SEM images. Based on these experiments, it can be stated that the merging process was further advanced in the HSM sample, resulting in a more firmly bound lubricant. The structure of the stirring tool and mixing vessel of the Picomix, as well as the longer processing time could be the reason. There might be less concentrated mechanical stress in the Picomix compared to the Picobond, but more particle–particle friction with the additive in the contact area could occur due to the more effective mixing. This suggests that the merging process was not yet complete in the Angmill Mechanofusion System, leading to a greater loss of residual, not firmly bound MgSt.

All samples were then also assessed in the SEA to enable an expressive comparison of BET surfaces after the coating process. The resulting BET surface areas are presented in Table 2. The AMS sample appeared very smooth after removal of residual MgSt, resulting in lower SSA. Even though no big changes were observable in SEM images for the HSM sample, the SSA had also decreased. Only the order of the SSA values was contradictory to the surface appearance, being lower for the sample with more visible small residual particles (Table 2). This could be due to a more irregular surface after the HSM coating, resulting in higher SSA, while the AMS sample surface area was more similar to the raw material IH230 SSA because of the insufficient coating mechanism.

Another technique, which was used to investigate the processing and coating process impact, was inverse gas chromatography. The measured surface energy can be plotted over the surface coverage. Since the surface energy is derived from chemical composition and physical properties, such as crystal habit, it is perfectly suitable for the investigation of particle coating processes. Prior to every measurement of surface energy, an octane adsorption isotherm was performed to calculate BET surface area (Table 2), which was then used to determine monolayer capacity. With known monolayer capacity, all injections of probe gases are comparable since specific surface coverages are injected instead of injecting fixed concentrations (leading to different surface coverages). In Table 4, the range of the different components of the surface energy of solids (i.e., dispersive and acid-base part) of the starting material IH230 and all processed materials are displayed. The total surface energy of solids (right column) is calculated as the sum of both parts. The surface energy is dependent on the surface coverage because at low surface coverages, the probe gases will primarily interact with high energy sites on the sample. With increasing surface coverage, more sites are taken into account. Usually, measurements would not be performed above 20% of the monolayer capacity because probe gas molecules start to interact with each other at higher concentrations [21].

**Figure 7:**
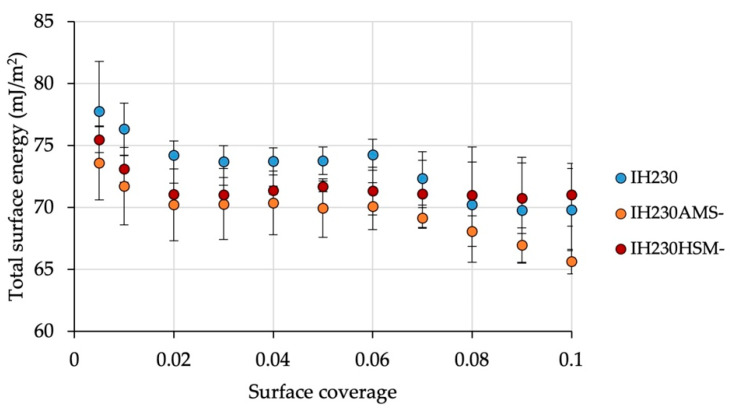
Total surface energy of the raw material in comparison to processed powders without the additive. *n* = 3, error bars show SD.

Comparing the surface energy distribution of the starting material with the material after processing without the additive, the dispersive part of the surface energy showed no significant changes. The acid-base (polar) part, however, decreased after processing. Considering the standard deviations, there was no significant change (*p*-values > 0.05 when comparing min. and max. values of the SE distribution). Figure 7 shows the SE distribution of the total surface energy measured for the starting material as well as the AMS and HSM samples without the additive. In combination with the measured BET surface area, it can be concluded that no significant surface alterations were caused solely by processing because these two methods cover the morphology part (surface roughness alteration would cause changes in BET SSA) as well as the crystal habit part (crystal habit alterations would lead to SE changes due to different adsorption behaviour in inverse gas chromatography).

**Figure 8:**
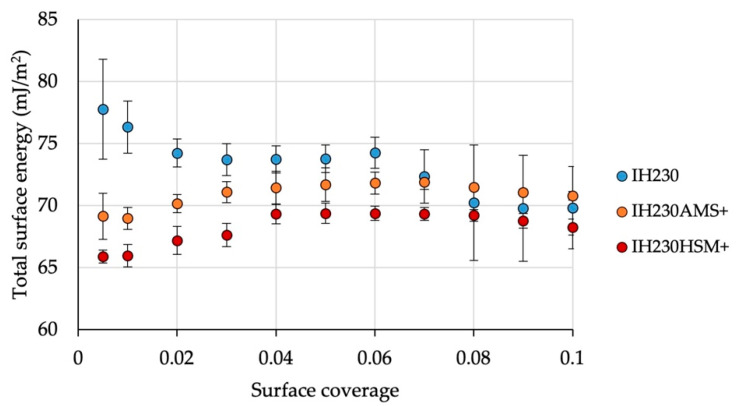
Total surface energy of the raw material in comparison to processed powders with the additive. *n* = 3, error bars show SD.

Processing with the model additive MgSt caused significant changes in surface area and surface energy (*p*-values < 0.05 when comparing surface area and surface energy maxima). At low surface coverages of the probe gases, the total surface energy decreased by up to 15.3% (HSM). Comparing both coating processes, it can be observed that coating in a high-shear mixer led to lower surface energies at low surface coverages (Figure 8), which could be linked to the levelling of higher energy sites. Measuring at higher surface coverages, the surface energies resemble one another (Table 4 and Figure 8). While the dispersive maximum of the SE shows no significant changes when comparing raw and processed materials, the polar part does. The maximum of the polar SE decreased by 13.3% (AMS) and 30.0% (HSM). This is a significant decrease (*p*-value < 0.05) as well as a significant difference between both processes (*p*-value < 0.005). Such decreases in surface energy can be linked to the decreased tendency of the surface to interact with surroundings (e.g., during adhesion) [22].

### 3.3. Assessment of Processed Powders as DPI Carriers

Decreases in interaction strength can therefore lead to significant changes in aerodynamic performance, as attachment and detachment of micronised drug particles will be altered. The impact of processing with the additive on resulting fine particle fractions (FPF) of the respective interactive blends assessed in the FSI is displayed in Table 5. The FPF increased after processing the carrier with MgSt by 128.2% (AMS) and 120.0% (HSM). The increases in comparison to the non-processed carrier were statistically significant (*p*-value < 0.001), but the difference between both processed carriers was not (*p*-value > 0.05). 

**Figure 9:**
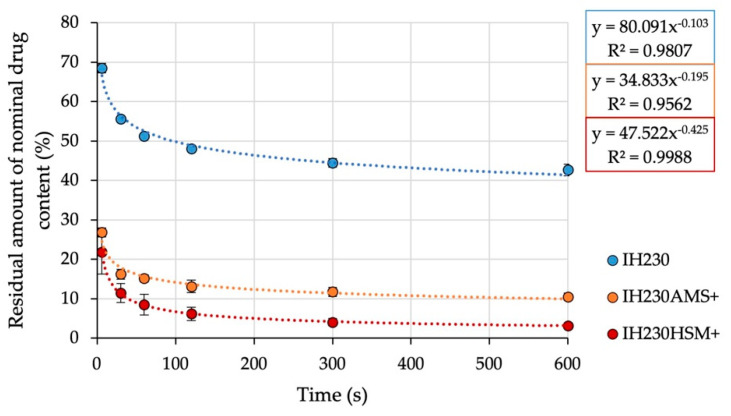
Residual amount of nominal drug content of respective interactive blends (percentage of nominal drug content) in dependence of the time dispersed by the AJS. *n* = 3, error bars show SD.

To gain further insight into the adhesion of drug particles on either non-processed or processed carriers, an adhesion force screening of the blends was carried out. The air-jet sieve (AJS) allows the simulation of a situation that is exemplary of a blend that is intended to be dispersed in the airstream. With a mesh size of 20 µm and a negative pressure of 4 kPa, mainly drug particles are sucked through the sieve and therefore eliminated from the dispersed (using pressurised air) system. Sampling at defined times and quantification of residual API in the blend allow conclusions of adhesion strength; the higher the adhesion strength, the less API will detach from the lactose particles. All prepared interactive blends were assessed with this method. The gathered data (Figure 9) were described with a power regression model (R^2^ > 0.95). The mean residual drug concentration (starting concentration defined as 100%) after 10 min of dispersing and removing particles below 20 µm is defined as carrier residue (CR). The slope of the linearised function describes the speed of decrease (SOD) of drug content in the dispersed system. In a real setting, the drug has to be detached in a split second [23] during inhalation. Thus, the SOD is a parameter allowing quantification of a formulation property with crucial influence on the performance. The CR however serves as a blend characteristic for comparing the share of the strongly bonded drug. The results are displayed in Table 6. 

All blends fulfilled the mentioned requirements to be considered homogenous before the testing, so the data point at zero seconds is 100% ± 10% (data point excluded to allow power regression). It can be observed that the HSM blend showed less adhesion forces between carrier and API, leading to higher detachment and drug content decreases in the dispersed system. The resulting CR and SOD values of all investigated blends differ in statistical significance (CR: *p*-values < 0.001; SOD: *p*-values < 0.05). This was confirmed by the results of the SEA analysis. Another indication for reduced adhesion between the carrier and drug was the share of drug deposited in the pre-separator of the FSI. It also represents an approximation of the amount of drug, which did not detach and, hence, impacted the lactose carrier in the pre-separator. The shares are shown in Table 7. They showed essentially the same trend as the AJS results, confirming higher drug detachment for the HSM blend. All differences were statistically significant (*p*-value < 0.001). 

## 4. Conclusions

Both high-shear mixing and mechanofusion were suitable for DPI carrier engineering. This study proves that dry particle coatings with specific additives (e.g., lubricants) lead to substantial alterations in particle behaviour. The investigation of coating attempts with and without coating materials allowed conclusions on the origin of the introduced changes. Using additive-free processes, the alterations in particle appearance, aerodynamic performance or adhesive properties were traced back to the additive, not the process. Even if AMS and HSM coatings both led to significant increases in FPF, the HSM coating showed superiority in terms of carrier residues and speed of detachment (of drug content). In addition, the SEM images before and after air-jet sieving showed a more complete coating for the HSM process. This was substantiated by the determination of firmly bound additive content. Even though SE decrease and therefore the decrease in the work of adhesion were superior for the HSM surface, the aerodynamic performance showed no significant difference between both options. This supports the theory of energetic levelling of higher energy binding sites on the DPI carrier surface. Although the AMS coating showed inferior coating efficacy, the high-energy sites on the lactose surface decreased for both coating strategies, which therefore substantiates the positive effect of using dry particle coatings in DPI formulation development. Furthermore, this work supports the hypothesis that adhesion strength between drug and carrier may be crucial but not solely decisive for the aerodynamic performance of the respective interactive blend. In conclusion, the HSM strategy should be preferred in a comparable experimental setup due to its reported benefits combined with less processing time.

## Figures and Tables

**Table 1:** Particle size distributions of all investigated materials. *n* = 3, SD in parentheses.

	IH230		IH230AMS−		IH230HSM−		IH230AMS+		IH230HSM+	
d_10_ (µm)	62.1	(0.1)	57.3	(0.5)	60.2	(0.1)	47.8	(1.7)	58.8	(0.7)
d_50_ (µm)	103.4	(0.1)	100.4	(0.4)	101.8	(0.2)	96.7	(0.6)	101.8	(0.2)
d_90_ (µm)	155.4	(0.1)	152.3	(0.5)	154.4	(2.0)	147.5	(0.9)	153.2	(0.2)
Span	0.9		1.0		0.9		1.2		0.9	

**Table 2:** SSA of the raw material and that processed with and without the additive. Data marked with “AJS” display results after removal of residual particles via air-jet sieving *n* = 3, SD in parentheses.

Octane BET SSA (m^2^/g)	IH230		IH230AMS		IH230HSM	
Processed without additive	0.105	(0.011)	0.110	(0.001)	0.106	(0.003)
Processed with additive			0.432	(0.020)	0.809	(0.102)
Processed with additive (AJS)			0.292	(0.010)	0.561	(0.010)

**Table 3:** Additive content before and after the removal of residual particles (AJS). *n* = 1.

	IH230AMS+	IH230AMS+ AJS	IH230HSM+	IH230HSM+ AJS
MgSt content (%)	1.31	0.52	1.23	1.01

**Table 4:** Results of the SE determination of the raw material in comparison to processed powders with and without the additive. *n* = 3, SD in parentheses.

	γ^D^ (mJ/m^2^)				γ^AB^ (mJ/m^2^)				γ^Total^ (mJ/m^2^)			
	Min		Max		Min		Max		Min		Max	
IH230	40.4	(0.8)	43.9	(1.3)	29.0	(3.7)	33.8	(2.3)	69.8	(4.2)	77.8	(3.6)
IH230AMS−	38.4	(0.7)	44.5	(0.5)	27.3	(1.1)	29.6	(2.6)	65.6	(1.0)	73.6	(3.0)
IH230HSM−	40.8	(1.2)	45.1	(0.2)	28.3	(1.8)	30.3	(1.7)	70.8	(2.1)	75.5	(1.0)
IH230AMS+	42.1	(0.7)	43.2	(0.6)	25.9	(1.3)	29.3	(0.9)	69.0	(1.3)	71.9	(0.7)
IH230HSM+	44.8	(0.9)	45.7	(0.9)	20.7	(0.4)	23.7	(0.3)	65.9	(0.7)	69.4	(0.7)

**Table 5:** FPF and FPD of interactive blends of all prepared adhesive mixtures. *n* = 5, SD in parentheses.

	IH230		IH230AMS+		IH230HSM+	
Fine Particle Fraction (%)	25.8	(1.4)	58.9	(1.4)	56.8	(2.5)
Fine Particle Dose (µg)	33.8	(1.5)	73.5	(2.0)	72.8	(5.0)

**Table 6:** Results of the AJS adhesion strength screening of all prepared adhesive mixtures. *n* = 3, SD in parentheses.

	IH230		IH230AMS+		IH230HSM+	
Carrier residue (%)	42.7	(1.5)	10.5	(0.9)	3.2	(0.2)
Speed of decrease	0.1	(0.01)	0.2	(0.02)	0.4	(0.07)

**Table 7:** Share of the recovered dose that impacted in the pre-separator of the FSI. *n* = 5, SD in parentheses.

	IH230		IH230AMS+		IH230HSM+	
Pre-separator impaction (%)	48.2	(2.9)	19.6	(0.9)	15.3	(1.2)

## Data Availability

Not applicable.

## References

[1] Labaki W.W., Han M.K. (2020). Chronic respiratory diseases: A global view. Lancet Respir. Med..

[2] Shur J., Price R., Lewis D., Young P.M., Woollam G., Singh D., Edge S. (2016). From single excipients to dual excipient platforms in dry powder inhaler products. Int. J. Pharm..

[3] De Boer A.H., Hagedoorn P., Hoppentocht M., Buttini F., Grasmeijer F., Frijlink H.W. (2017). Dry powder inhalation: Past, present and future. Expert Opin. Drug Deliv..

[4] Steckel H., Müller B.W. (1997). In vitro evaluation of dry powder inhalers I: Drug deposition of commonly used devices. Int. J. Pharm..

[5] Jones M.D., Price R. (2006). The Influence of Fine Excipient Particles on the Performance of Carrier-Based Dry Powder Inhalation Formulations. Pharm. Res..

[6] Guchardi R., Frei M., John E., Kaerger J. (2008). Influence of fine lactose and magnesium stearate on low dose dry powder inhaler formulations. Int. J. Pharm..

[7] Hertel N., Birk G., Scherließ R. (2020). Performance tuning of particle engineered mannitol in dry powder inhalation formulations. Int. J. Pharm..

[8] Jetzer M.W., Schneider M., Morrical B.D., Imanidis G. (2018). Investigations on the Mechanism of Magnesium Stearate to Modify Aerosol Performance in Dry Powder Inhaled Formulations. J. Pharm. Sci..

[9] Tay T., Das S., Stewart P. (2010). Magnesium stearate increases salbutamol sulphate dispersion: What is the mechanism?. Int. J. Pharm..

[10] Nicholas M., Josefson M., Fransson M., Wilbs J., Roos C., Boissier C., Thalberg K. (2020). Quantification of surface composition and surface structure of inhalation powders using TOF-SIMS. Int. J. Pharm..

[11] Kumon M., Suzuki M., Kusai A., Yonemochi E., Terada K. (2006). Novel Approach to DPI Carrier Lactose with Mechanofusion Process with Additives and Evaluation by IGC. Chem. Pharm. Bull..

[12] Hosokawa Micron Ltd. Picobond Product Description. https://www.hosokawa.co.uk/products/picobond/.

[13] Das S.C., Zhou Q., Morton D.A., Larson I., Stewart P.J. (2011). Use of surface energy distributions by inverse gas chromatography to understand mechanofusion processing and functionality of lactose coated with magnesium stearate. Eur. J. Pharm. Sci..

[14] Jetzer M.W., Morrical B.D., Schneider M., Imanidis G. (2016). Investigating the Effect of the Force Control Agent Magnesium Stearate in Fluticasone Propionate Dry Powder Inhaled Formulations with Single Particle Aerosol Mass Spectrometry (SPAMS). Drug Deliv. Lungs.

[15] Sharma R., Setia G. (2019). Mechanical dry particle coating on cohesive pharmaceutical powders for improving flowability—A review. Powder Technol..

[16] Zhou Q., Qu L., Gengenbach T., Larson I., Stewart P.J., Morton D.A.V. (2012). Effect of Surface Coating with Magnesium Stearate via Mechanical Dry Powder Coating Approach on the Aerosol Performance of Micronized Drug Powders from Dry Powder Inhalers. AAPS PharmSciTech.

[17] Das S.C., Stewart P.J. (2012). Characterising surface energy of pharmaceutical powders by inverse gas chromatography at finite dilution. J. Pharm. Pharmacol..

[18] Ylä-Mäihäniemi P.P., Heng J.Y.Y., Thielmann F., Williams D.R. (2008). Inverse Gas Chromatographic Method for Measuring the Dispersive Surface Energy Distribution for Particulates. Langmuir.

[19] Kondor A., Williams D.R., Burnett D.J. Determination of Acid-Base Component of the Surface Energy by Inverse Gas Chromatography: iGC SEA Application Note 227. https://www.surfacemeasurementsystems.com/downloads/sea-application-notes/.

[20] Kaialy W., Alhalaweh A., Velaga S.P., Nokhodchi A. (2012). Influence of lactose carrier particle size on the aerosol performance of budesonide from a dry powder inhaler. Powder Technol..

[21] Kondor A., Burnett D.J., Williams D.R. Infinite Dilution and Surface Energy Heterogeneity Profile by iGC-SEA; iGC-SEA Technical Note 807. https://www.surfacemeasurementsystems.com/downloads/sea-application-notes/.

[22] Williams D.R. (2015). Practicle Engineering in Pharmaceutical Solids Processing: Surface Energy Considerations. Curr. Pharm. Des..

[23] Behara S.R.B., Kippax P., Larson I., Morton D.A., Stewart P. (2011). Kinetics of emitted mass—A study with three dry powder inhaler devices. Chem. Eng. Sci..

